# Self-Assembly in Ganglioside‒Phospholipid Systems: The Co-Existence of Vesicles, Micelles, and Discs

**DOI:** 10.3390/ijms21010056

**Published:** 2019-12-20

**Authors:** Enamul Haque Mojumdar, Carl Grey, Emma Sparr

**Affiliations:** 1 Physical Chemistry, Lund University, 221 00 Lund, Sweden; 2 Division of Biotechnology, Lund University, 221 00 Lund, Sweden; carl.grey@biotek.lu.se

**Keywords:** ganglioside GM1, phospholipids, micelles, vesicles, lipid discs, polarization transfer solid state NMR (PTssNMR), cryo-transmission electron microscopy, small-angle X-ray scattering

## Abstract

Ganglioside lipids have been associated with several physiological processes, including cell signaling. They have also been associated with amyloid aggregation in Parkinson’s and Alzheimer’s disease. In biological systems, gangliosides are present in a mix with other lipid species, and the structure and properties of these mixtures strongly depend on the proportions of the different components. Here, we study self-assembly in model mixtures composed of ganglioside GM1 and a zwitterionic phospholipid, 1,2-Dioleoyl-sn-glycero-3-phosphocholine (DOPC). We characterize the structure and molecular dynamics using a range of complementary techniques, including cryo-TEM, polarization transfer solid state NMR, diffusion NMR, small-angle X-ray scattering (SAXS), dynamic light scattering (DLS), and calorimetry. The main findings are: (1) The lipid acyl chains are more rigid in mixtures containing both lipid species compared to systems that only contain one of the lipids. (2) The system containing DOPC with 10 mol % GM1 contains both vesicles and micelles. (3) At higher GM1 concentrations, the sample is more heterogenous and also contains small disc-like or rod-like structures. Such a co-existence of structures can have a strong impact on the overall properties of the lipid system, including transport, solubilization, and partitioning, which can be crucial to the understanding of the role of gangliosides in biological systems.

## 1. Introduction

Ganglioside lipids have been associated with several physiological processes, including cell signaling, neuronal recovery and protection, and apoptosis [1,2,3]. They are highly present in the nervous system, where they account for ~15 % of the total lipid mass in the neuronal membrane [4,5,6,7], and they have also been identified in cell-derived vesicles, for example, exosomes [8]. We have previously demonstrated that ganglioside-containing exosomes, as well as vesicles prepared from extracted exosome lipids and ganglioside-containing model mixtures, accelerate the amyloid formation of α-synuclein, which is related to Parkinson’s disease [8]. Ganglioside lipids have also been proposed to be relevant for other diseases, for example, Alzheimer’s disease, Tay‒Sachs disease, and cancer [9,10,11,12,13,14,15,16].

Ganglioside lipids were first described by Klenk for lipids newly isolated from ganglion cells in the brain [17,18,19]. They belong to the glycosphingolipids family and are composed of a ceramide and oligosaccharide backbone, with one or more sialic acids [4]. Depending on the number of sialic acid residues linked to the sugar backbone, gangliosides (G) can be mono (M), di (D), tri (T) sialylated and abbreviated as GM, GD, GT, etc. according to the Svennerholm nomenclature [20,21]. The GM class are further categorized into GM1, GM2, and GM3, which differ in the number of sugar residues present in the oligosaccharide backbone. There are variations in ganglioside composition between individuals and tissues, although GM1 is considered to be the most common ganglioside in healthy non-neural tissues [6,22]. In neural cells, GM gangliosides are expressed together with the more sialylated GD and GT gangliosides [6,23].

**Figure 1:**
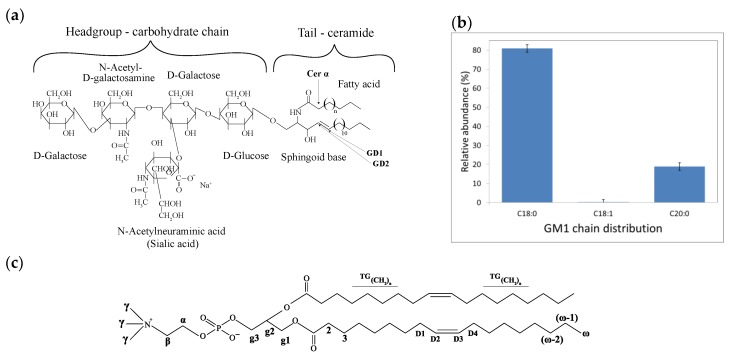
(**a**) Molecular structure of ganglioside GM1. The large oligosaccharide headgroup of GM1 consists of five sugar residues and the tail ceramide part is composed of a sphingoid base and variable fatty acyl chains that are indicated in the structure. (**b**) Relative abundance ± standard deviation of the chain length distribution of ovine GM1 obtained from nano-ESI-MS measurements. (**c**) Molecular structure of DOPC. The different molecular segments in the head-group and in the acyl chains of DOPC and GM1 (**a**,**c**) are labeled here to support the NMR peak assignments.

The ganglioside lipid studied here, GM1, contains four sugar residues in the oligosaccharide backbone (Figure 1a). The large oligosaccharide with negatively charged sialic acid moieties in the GM1 head-group gives the GM1 molecule a conical shape [24], thus promoting the formation of nonlamellar structures with positive curvature, including micelles, cubic micellar, and cubic bicontinuous phases [25]. In biological systems, gangliosides are primarily found in the outer plasma membrane, and they are present at relatively high concentrations (up to ca. ~15 mol % in neurons [4,6,7]). The gangliosides are soluble to some extent in the planar lipid bilayer. Numerous studies have shown that gangliosides have a condensing effect in lipid bilayers, leading to increased acyl chain order [26,27,28]. Gangliosides have also been associated with the formation of nanodomains (compare so-called rafts) in cell membranes [29,30], which have also been associated with membrane‒protein interactions [31,32] as well as membrane-induced amyloid formation [8,33,34].

In summary, the GM1-phospholipid self-assembly structure strongly depends on the proportions of the different lipid components in the mixture. In GM1-rich systems, (mixed) micelles will form, while planar bilayers will be formed in phospholipid-rich systems. On thermodynamic grounds, it follows that for GM1-phospholipid mixtures with compositions in between those extreme cases where only one type of structure is present, the sample will be heterogenous and contain two or more co-existing structures. Such heterogeneity will have a strong impact on the overall properties of the lipid system, including the transport properties, solubilization of hydrophobic species, partitioning of interacting molecules between the different assemblies, relaxation rates, aggregate curvature, etc. Still, this co-existence in ganglioside-containing systems is not well characterized in the literature, and this is the focus of this paper.

We here investigate model systems composed of ovine brain extract of GM1 and 1,2-dioleoyl-sn-glycero-3-phosphocholine (DOPC) at different molar ratios of DOPC-GM1. First, we show that GM1 has a strong condensing effect on the acyl chains in mixed lipid bilayers, which is in line with previous reports. We also confirm that GM1 alone in water forms micelles. Then we focus on characterizing the heterogenous samples that contain different types of co-existing structures using a range of complementary physical-chemical techniques, including nuclear magnetic resonance (NMR), transmission electron microscopy (TEM), small-angle X-ray scattering (SAXS), light scattering, and calorimetry. It is a major conclusion that one reaches the conditions where the bilayer co-exists with other smaller dispersed micellar structures at low GM1 concentrations. This co-existence is highly relevant in terms of physiological ganglioside contents.

## 2. Results

Thanks to intermolecular interactions, the mixing of different lipid species may lead to the formation of new structures with altered properties that cannot be achieved by the single components. Here we aim at the characterization of the structure and molecular dynamics of mixed lipid systems containing GM1 and DOPC (Figure 1a,c). We first investigate the changes in the lipid molecular dynamics upon mixing these two types of lipid species. It is shown that the lipid acyl chains are clearly more rigid in the mixtures than for either of the two lipids alone. We then demonstrate, using cryo-TEM, that the mixed system has a rich phase behavior with several co-existing self-assembled structures. GM1, alone in an aqueous solution, forms micelles, while DOPC alone forms lipid bilayers. In the mixed system, these structures are present, as well as additional disc-like objects. The mixed DOPC-GM1 systems are then studied using bulk scattering and NMR techniques. The acyl chain composition of the GM1 extract was analyzed using nano-electrospray ionization mass spectrometry (nano-ESI-MS), showing that the dominant chain component is C18:0, and that the sample also contains gangliosides with a C20:0 chain (Figure 1b). All experiments were performed in 10 mM MES buffer pH 5.5. The sialic acid residue of GM1 has a pKa of ca. 2.7 [35] and the ganglioside headgroup is therefore expected to carry one negatively charge for the present pH conditions as well as neutral conditions. The mildly acidic pH is relevant to some cellular environments, such as endosomes and lysosomes. At these conditions, ganglioside-containing membranes have also been shown to trigger the aggregation of the amyloid protein α-synuclein, which is associated with Parkinson’s disease [8,36]. For practical reasons, the overall lipid concentration varies between experiments using different methods, so an overview of the concentrations and molar ratios in all samples investigated is given in Table 1.

### 2.1. Lipid Molecular Dynamics in DOPC-GM1 Mixtures

We here study how the lipid molecular dynamics changes for different ratios of DOPC-GM1 using polarization transfer solid-state NMR (PTssNMR) on natural abundance 13C [37]. These experiments provide information on the mobility and conformation in different segments of the lipid molecules with close-to-atomic resolution [37,38]. The same experimental approach has previously been used to yield atomically resolved information on molecular dynamics in lipids and surfactants [38,39] and in amyloid fibrils [40,41], as well as in samples with a complex composition from lung surfactant [42], cartilage [43], and skin stratum corneum [44]. Three different NMR experiments were performed for each sample: DP (direct polarization), CP (cross-polarization), and INEPT (insensitive nuclei enhanced by polarization transfer). The CP and INEPT experiments rely on ^1^H to ^13^C polarization transfer, while the DP record pulses from all ^13^C in the sample and is used as a reference. In the CP experiments, polarization transfer occurs through space dipolar couplings, and in the INEPT experiments the polarization transfer occurs via bond couplings. Therefore, CP is most effective at boosting signals for segments with slow and/or anisotropic motion. INEPT, on the other hand, is highly efficient at boosting signals for segments with fast and/or isotropic motion. When we only detect INEPT signals without a CP contribution, we could define that motion as “isotropic”. A contribution of both INEPT and CP would lead us to define the molecular motion as “anisotropic”.

**Figure 2:**
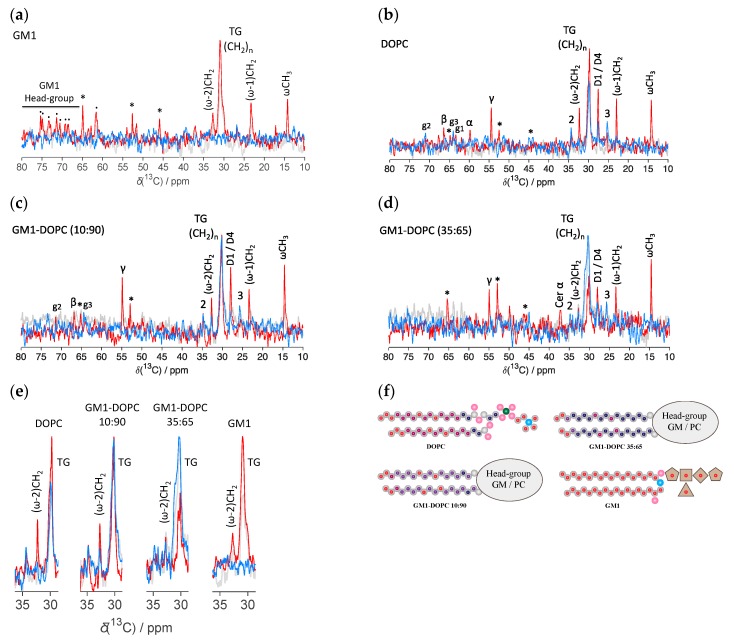
PTssNMR ^13^C spectra of lipid-water mixtures (**a**) GM1 (**b**) DOPC, (**c**) DOPC-GM1 molar ratio 90:10 and (**d**) DOPC-GM1 molar ratio 65:35 at 37 °C. The individual DP (gray), CP (blue), and INEPT (red) spectrum are overlaid and the peaks are assigned to different lipid molecular segments and labeled according to Figure 1c. The asterisk (*) indicates resonance lines originating from the MES buffer. The dot (**.**) in the INEPT spectrum indicates peaks originating from the oligosaccharide headgroup of the GM1 molecule. The peaks from the oligosaccharide headgroup are not resolved for the mixed DOPC-GM1 system due to the low GM1 concentration. (**e**) Close-up view of the main chain (CH_2_)_n_ TG peak at approx. 30.5 ppm for various lipid mixtures, showing how the relative signal intensities of CP and INEPT vary for samples with different GM1 contents. (**f**) Different carbon moieties in lipid acyl chains of GM1 and DOPC molecules that exist in different dynamic regimes, as revealed by PTssNMR. Red = carbons, for which an INEPT signal is detected, indicating fast isotropic motion. Navy blue = carbon segments that exhibit a strong CP signal together with a low INEPT signal, indicating “anisotropic” motion. The maroon and purple indicate that both INEPT and CP signals are detected and that the INEPT signal is higher compared to the navy blue labeled carbon, implying higher mobility. The INEPT/CP ratio increases in the order navy blue < purple < maroon. The total lipid concentration was 15 mM for the sample containing only GM1, and 25 mM for DOPC-containing samples.

Figure 2 shows the PT ssNMR spectra for samples composed of GM1 and DOPC, as well as their mixtures (molar ratio of DOPC:GM1 = 90:10 and 65:35). In all figures, the individual DP (gray), CP (blue), and INEPT (red) spectra are overlaid and the different molecular segments are labeled according to Figure 1a,c. An asterisk (*) indicates the resonance lines originating from the MES buffer (Figure S1a). A dot (.) in the INEPT spectrum represents peaks originating from the oligosaccharide headgroups of the GM1 molecule. The crowded spectral region around 30.5 ppm is indicative of the acyl chain trans/gauche (TG) conformations, which is expected for a fluid bilayer phase. This can be distinguished from the all-trans (AT) conformation that would be expected for a solid (gel phase) bilayer, which gives rise to a peak centered around 33.5 (AT) ppm [42]. For a system with co-existing solid and fluid domains, one should expect to distinguish both AT and TG crowded spectra regions [42,45].

For the sample consisting of GM1 in buffer (Figure 2a), only an INEPT signal is observed for the whole spectral regime, and there are no visible CP signals from any segment in the GM1 molecule. This is consistent with the formation of micelles with a fluid interior and isotropic motion. On the other hand, the PTssNMR spectra for the samples composed of DOPC alone show both CP and INEPT resonance lines (Figure 2b), which is consistent with anisotropic motion in a liquid crystalline phase, for example the lamellar phase [38]. The peak around 30.5 ppm is indicative of TG conformation in the acyl chains.

**Figure 3:**
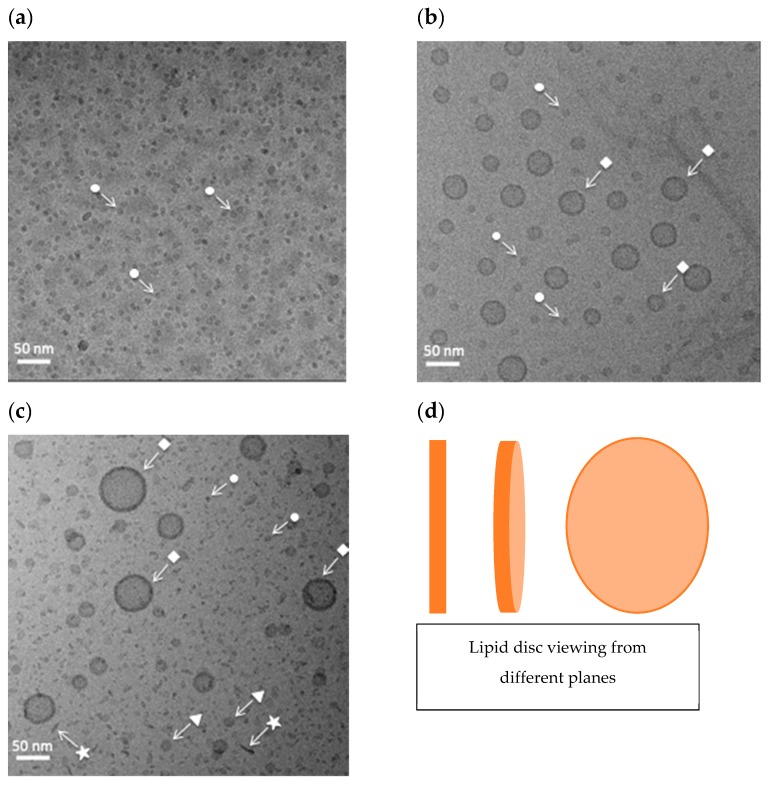
Representative cryo-TEM images for systems containing (**a**) GM1, (**b**) DOPC-GM1 molar ratio 90:10, and (**c**) DOPC-GM1 molar ratio 65:35. In the sample containing only GM1 (**a**), micellar aggregates with a diameter above 8 nm are observed (white arrow with circle). In the mixed DOPC-GM1 systems (**b**,**c**), unilamellar vesicles (square symbol) co-exist with either micellar aggregate (circle symbol) at the lower GM1 content (**b**). At higher GM1 contents (**c**), the sample is more polydisperse, containing different types of small objects, which are either interpreted as distorted micelles (circle symbol) or disc-like objects viewed from different angles (triangle and star symbol), as illustrated in (**d**). All samples were prepared in 10 mM MES buffer pH 5.5, and the total lipid composition was 15 mM (**a**) or 25 mM (**b**,**c**).

It is striking that for samples that contain both DOPC and GM1 mixtures (Figure 2c,d), the intensity of the INEPT/CP and INEPT/DP ratios are drastically reduced as compared to the spectra from the individual components (Figure 2a,b). This is shown in more detail in Figure 2e, which zooms in on the spectral regime around the main chain (CH_2_)_n_ TG peak. A schematic illustration of how the molecular dynamics vary within the lipid acyl chains for different DOPC-GM1 ratios is given in Figure 2f. From these experiments, we can conclude that the addition of GM1 to the phospholipid system leads to slower average dynamics or an increased order parameter of the lipid acyl chains as compared to the pure DOPC or GM1 systems. This is consistent with previous reports that GM1 has a condensing effect in phospholipid systems [26,27,28]. We only observe a signal from the TG chain conformation and there are no indications of a co-existing lipid population with AT conformation, as would be expected in the case of the segregation of solid (gel phase) and fluid bilayer domains. The same trends are also seen for the (ω-2) carbons (Figure 2e) and for D2/D3 unsaturated double bonds (Figure S1b). We note that the signal from the acyl chains in DOPC and GM1 cannot be distinguished for carbon segments ω, (ω-1), (ω-2), and (CH_2_)_n_ TG. The resonances from the GM1 headgroup cannot be resolved in the sample with only 10 mol % GM1. For higher GM1 concentrations, the INEPT resonance line from ceramide α is observed in the INEPT spectra at 37 ppm (Figure 3d), suggesting that a fraction of GM1 headgroups also exhibit isotropic motion in the mixed system. Similar results were obtained when the same experiments were performed at 32 °C (Figure S2).

In summary, the NMR data show that there is reduced chain mobility in the DOPC-GM1 mixtures compared to systems containing only GM1 or DOPC. However, these NMR experiments cannot discriminate what type of aggregates are formed. For a system with fast exchange, we observe an average signal coming from all carbons of the same type in the whole sample, and cannot distinguish whether these chains are present in the same type of aggregates, or if we observe an average property from the acyl chains present in different types of aggregates. This motivates further investigations of the self-assembly structures in the very same systems.

### 2.2. Cryo-TEM Observations of DOPC-GM1 Mixtures

As a next step, we use cryo-TEM to image samples with the same composition as those studied with PTssNMR (Figure 3). The aqueous dispersions were rapidly frozen, thus providing information on the structure in solution. Additional images of the same samples are shown in Figure S3. In the sample that contains only GM1 in buffer, small and rather monodisperse objects are seen (white arrow with filled circle) (Figure 3a), interpreted as GM1 micelles in accordance with previous reports [46,47]. When GM1 is mixed with DOPC, the samples contain more than one type of structures, so unilamellar vesicles (the square symbol) co-exist with smaller objects that vary in size and shape depending on the GM1 content. In the sample that contains 10 mol % GM1 (Figure 3b), the vesicles co-exist with globular objects, similar to the micelles seen for the GM1 system (Figure 3a). At higher GM1 contents (DOPC-GM1 65:35, Figure 3c), the vesicles co-exist with polydisperse objects of varying size and shape. The smallest objects are similar to the micelles seen in the pure GM1 system, although they seem slightly distorted. The sample also contain slightly bigger objects that have both round (triangle symbol) and rod-like (star symbol) assemblies. The contrast of the round objects in the electron beam is rather homogenous across the object, without a high-contrast edge as is seen for the vesicles. The diameter of these round objects is similar to the length of the rods. Based on these observations, we propose that these rod-like and round structures originate from the same type of disc-like objects that are seen from different directions (Figure 3d). For the system composed of DOPC alone, it is well established that one can form unilamellar vesicles after the sonication of a lipid dispersion [48,49].

The sample with the highest GM1 content (Figure 3c) contains more than two types of objects. This is not expected on thermodynamic grounds for an equilibrium binary lipid sample in excess water (unless one happens to perform the experiment at a temperature that exactly corresponds to a three-phase line in the phase diagram). In this context, it is pointed out that the commercial GM1 sample is not one single component, but an ovine brain extract that may contain a mixture of GM1 species (Figure 1b).

From the cryo-TEM images, we obtain estimates of the size of the different objects in the samples. The diameter of the micelles formed by GM1 alone (Figure 3a) was measured to be 8‒9 nm, while the diameter of the micelles observed in the samples of DOPC-GM1 molar ratio 90:10 (Figure 3b) are slightly larger, ca. 10 nm. The smallest distorted objects in the samples composed of DOPC-GM1 molar ratio 65:35 were measured to have a diameter of 7‒9 nm. From the MS analysis, we know that most GM1 molecules contain 18 or 20 carbons, and the fully-extended acyl chains thus have a length of ca. 2.0 nm ($l=0.15+0.12\;\times n_{c}$, $n_{c}$=18–20) [50]. The GM1 headgroup is five residue branch oligosaccharides. Given the length of a glucose molecule, approx. 0.8 nm in the long axis [51], the length of the GM1 oligosaccharide headgroup can be estimated at roughly 2.5‒3.0 nm. This gives rise to a total estimated length of the fully stretched GM1 lipid molecule of around 4.5‒5.0 nm. The diameter of the micelle is expected to be roughly twice the length of the molecule [50]—that is, around 9‒10 nm. This estimation is consistent with the observed size of GM1 micelles in the cryo-TEM images in Figure 3a‒c, with measured diameters in the range of 8‒10 nm. The diameter of the proposed disc-like structures was estimated from the cryo-TEM images (Figure 3c) to be 15‒20 nm.

In summary, it is clear from the cryo-TEM images (Figure 3 and Figure S3) that the mixed DOPC-GM1 samples not only form vesicles, but also other smaller objects. Based on the differences in molecular shape of the DOPC and GM1 molecules, we predict that the smaller objects are rich in GM1, and the vesicles are rich in DOPC. For the lipid mixtures, we also observe disc-like or rod-like structures that are not present for the systems containing only DOPC or GM1.

### 2.3. Scattering and NMR Characterization of GM1 Micelles and DOPC-GM1 Mixtures

**Figure 4:**
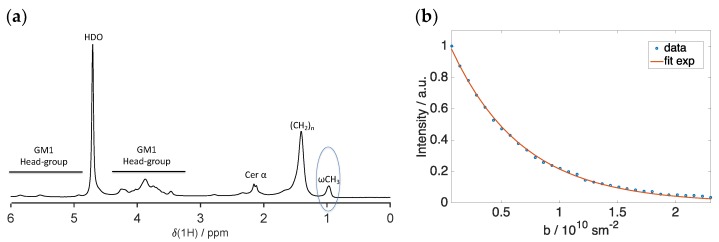
(**a**) ^1^H NMR spectrum of 15 mM GM1 measured at 37 °C. The signals arising from the GM1 lipid tails and from the headgroups are labeled in the spectrum. The peak at 0.9 ppm (marked with blue circle), corresponding to ωCH_3_, was used for measuring the diffusion coefficient. (**b**) Stejskal-Tanner plot showing the intensity of ωCH_3_ peak versus magnetic field strength *b* in the pulse field gradient stimulated echo (PFG-STE) diffusion experiments of 15 mM GM1. The solid line represents the single exponential fit to the experimental data points. The individual dots in the plot indicate intensity values obtained experimentally, and the solid line represents the mono exponential fit to the data points.

The GM1 micellar system was characterized using DLS, diffusion NMR, and SAXS. The scattering techniques DLS and SAXS provide information on particle size distribution, and through fitting of the SAXS data one can also obtain information on particle shape. The total lipid concentration varied a bit between these experiments for technical reasons (Table 1). From DLS studies on the GM1 micelles, we conclude that there is a rather monodisperse distribution of particle size, with a mean diameter of ca. 8 nm (Figure S4a). The size measurement from DLS is consistent with the finding from the cryo-TEM studies (Figure 3a). From ^1^H NMR self-diffusion experiments, we can further determine the average diffusion coefficient of ganglioside molecules in the aqueous solution, which in turn will depend on the size of the self-assembled GM1 aggregates. Figure 4 shows the ^1^H NMR spectrum of GM1 in pure water (Figure 4a) and the Stejskal-Tanner plot for the ωCH_3_ peak measured at various gradient strengths, where a solid line represents a single exponential fit to the experimental data points (Figure 4b). From this experiment, we obtain a diffusion coefficient (*D*) of 1.04 × 10^−10^ m^2^/s for the GM1 micelles (15 mM GM1 in buffer). Assuming that all GM1 molecules are present in the spherical micellar aggregates, the micelle diameter can be estimated at ~6‒7 nm using the Stokes-Einstein equation, $D=\frac{kT}{6\pi \eta R}$, where *R* is the hydrodynamic radius, k is the Boltzmann constant, *T* is the absolute temperature, and η is the dynamic viscosity. This slight discrepancy between the results obtained by diffusion NMR and DLS results may be related to the differences in lipid concentration in the samples (Table 1). It is also noted that for a system with fast exchange, the measured diffusion coefficient is a weighted average of the diffusion of monomers and micelles, and there is a slight underestimation of the micelle diffusion (and size), as the analysis presented above does not account for the free monomers. However, we judge this effect to be rather small, as the critical micelle concentrations (CMC) of GM1 are very low (1–80 μM according to previous reports [52,53,54,55]).

**Figure 5:**
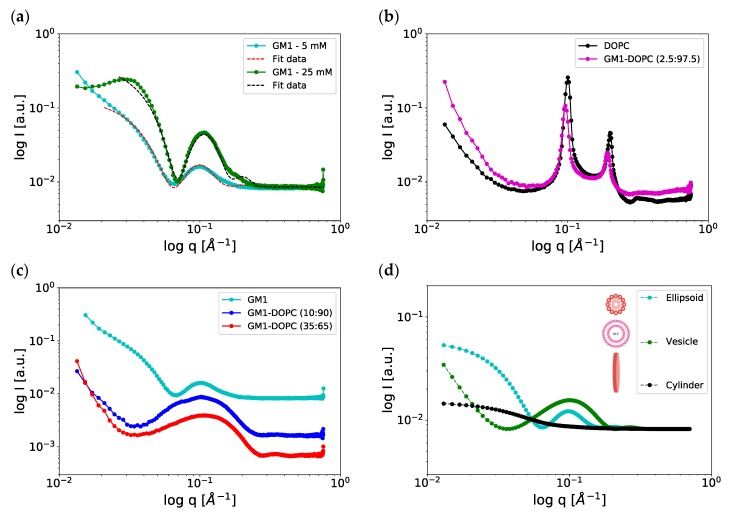
(**a**) SAXS curves *I(q)* of ganglioside GM1 at two different concentrations, 25 mM (green) and 5 mM (cyan), measured at 37 °C. The corresponding fit using a core shell ellipsoid model is also presented (dashed lines). (**b**) SAXS patterns of DOPC only (black) and DOPC-GM1 mixture with molar ratio 97.5:2.5 (magenta). (**c**) SAXS patterns of DOPC-GM1 mixtures with higher amounts of GM1, molar ratios 90:10 (blue) and 65:35 (red). As a reference, data for the 5 mM GM1 sample (cyan) are also included in the figure. The concentration for all DOPC-GM1 mixture samples is 25 mM. All the SAXS patterns were recorded at 37 °C. (**d**) Simulated SAXS patterns for different types of relevant objects, including core shell ellipsoid (cyan), vesicle (green), and short cylinder (compare disc) (black). The contrast in the SAXS patterns for DOPC-GM1 mixed systems is dominated by vesicles, as seen from the simulated patterns for ellipsoid, vesicle, and cylinder.

Next, we investigated samples composed of GM1 alone or mixtures of DOPC-GM1 using SAXS. Figure 5a shows the SAXS patterns obtained from samples composed of GM1 in buffer solution at two different concentrations, 25 mM (green) and 5 mM (cyan). The SAXS pattern from the sample with the higher concentration shows an evident minimum at *q* ≈ 0.06 Å^−1^, followed by a broad peak centered at *q* ≈ 0.12 Å^−1^. This suggests that the system contains globular objects. The data were fitted using a core-shell ellipsoid model to calculate the size of the micelles. Here, the core corresponds to the GM1 acyl chains, which have a lower contrast compared to the shell that corresponds to the solvated large polysaccharide headgroups. Using fitting parameters (radius of the core, shell thickness, and axial ratios polar to equatorial radii), the size of the micelles is estimated to be 9 nm and 10 nm in the polar and equatorial planes. Since the axial ratio is < 1, the shape of the micelles is slightly oblate spheroid and very close to spherical objects. In the same SAXS spectrum for the sample with the highest concentration, there is an additional peak at *q* ≈ 0.04 Å^−1^, which is likely attributed to the intermicellar interactions originating from electrostatic and entropic steric repulsion between the headgroups [56]. Based on the position of this peak, one can obtain a measurement of the apparent nearest neighbor intermicellar distance (*d*), which was here calculated as ca. 160 Å (*d* = $\frac{2\pi }{q}$). The corresponding peak is not detected for the more dilute sample. Furthermore, the minimum in the SAXS curve was slightly shifted to a lower *q* value at the lower GM1 concentration, indicating slightly larger micelles at the lower GM1 concentration. The estimated size of the micelles from SAXS fitting (diameter ca. 10 nm) is consistent with the cryo-TEM, DLS, and diffusion NMR experiments at similar lipid concentrations. Taken together, the combination of data demonstrates micellar aggregates with a slightly distorted shape in the aqueous solution.

From the cryo-TEM studies (Figure 3), we know that DOPC-GM1 mixtures contain a number of different self-assembled structures. Figure 5b,c shows the SAXS spectra for DOPC-GM1 mixtures with varying GM1 contents. These experiments were also performed for samples with the same lipid composition but neutral pH, showing very similar results (Figure S5). The sample composed of DOPC with no added GM1 (Figure 5b—black curve) shows characteristic Bragg peaks, indicative of a lamellar phase with a repeat distance (*d*) of ~60 Å. This is in close agreement with previous reports for DOPC lamellar phase in pure water [57]. The addition of a small amount of GM1 (2.5 mol %) leads to a slight shift of Bragg reflections towards lower *q* values, implying an increase in the lamellar repeat distance to 64 Å (Figure 5b—magenta curve). This increase can be understood on the basis of the introduction of an anionic GM1 lipid into the bilayer, leading to both electrostatic repulsion and entropic repulsion between the bilayers. When the ganglioside content is further increased to 10 mol % (Figure 5c—blue curve), which corresponds to the sample in the cryo-TEM image in Figure 3b, the SAXS pattern is clearly altered and shows only one broad hump centered around *q* ≈ 0.1 Å^−1^, with a minimum at *q* ≈ 0.03 Å^−1^. This suggests spontaneous formation of smaller dispersed objects without detectable multilamellar structures. Further addition of GM1 to 35 mol % shifts the minimum and the broad hump slightly to higher *q* values (Figure 5c—red curve). This indicates polydispersity and a change in shape or size of the objects present in the system [46,56,58] We could not obtain a proper fit of these SAXS patterns using only a core-shell model for vesicle. From the cryo-TEM images in Figure 3, we know that these samples contain several different types of objects. These give rise to slightly different scattering patterns; some examples of the simulated data are illustrated in Figure 5d. In a sample with several different types of structures, we will have an overlay of these scattering patterns, and such fitting would have too many fitting parameters to be meaningful. Finally, the DLS measurements for these lipid mixtures show a broad size distribution, which is also consistent with the interpretation of co-existing structures in the sample (Figure S4b).

**Figure 6:**
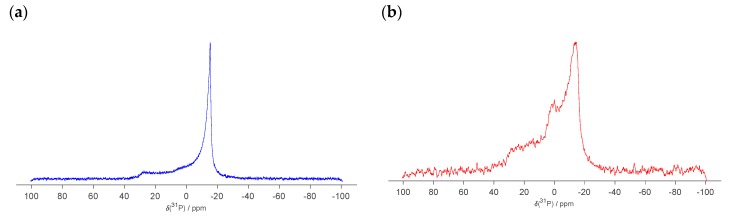
^31^P NMR spectra of 25 mM DOPC-GM1 mixtures at molar ratios of (**a**) 90:10 and (**b**) 65:35. Due to the short measurement time, the spectrum looks noisy for the 65:35 mixture. The measurements were performed at 37 °C.

Finally, we examined the same DOPC-GM1 samples as studied with SAXS using static phosphorus NMR (Figure 6). As phosphorus is only present in DOPC and not in GM1, we selectively study how the phospholipids distribute between the different self-assembly structures. The chemical shift anisotropy (CSA) is the difference in ppm between the high field peak and the shoulder in the low field region. For DOPC-GM1 lipid mixtures, the effective CSA is approximately 40 ppm, which is consistent with the CSA observed for PC phospholipids in fluid lamellar phase [59,60,61,62,63]. In addition, a sharp peak at a chemical shift close to 0 ppm is observed for the sample composed of DOPC-GM1 65:35 (Figure 6b), which implies that a fraction of DOPC is present in a co-existing isotropic object with fast tumbling. These small objects can be micelles, discs, or very small size unilamellar vesicles [59,60,63]. The corresponding isotropic peak is less evident in the spectra from the DOPC‒GM1 90:10 mixture (Figure 6a), which might be due to the lower GM1 content. In summary, using ^31^P NMR, we again observed that DOPC‒GM1 mixtures contain mixed objects, which is consistent with the SAXS and cryo-TEM findings.

### 2.4. Temperature-Induced Changes in the GM1-Water System

The PT ssNMR studies show that the addition of gangliosides rigidifies the acyl chains in the mixed lipid bilayers, as inferred from the increased CP signal (Figure 2). Such an ordering effect of gangliosides have also been proposed in previous studies [26,27,28,64]. This may be due to the strong interactions between the specific lipid components, or it could indicate that one is close to the solid‒fluid phase transition. We therefore investigated the possibility of temperature-induced transitions in the present bovine extract GM1 with mainly C18:0 and C20:0 chains (Figure 1b) using DSC, SAXS, and PTssNMR (Table 1).

**Figure 7:**
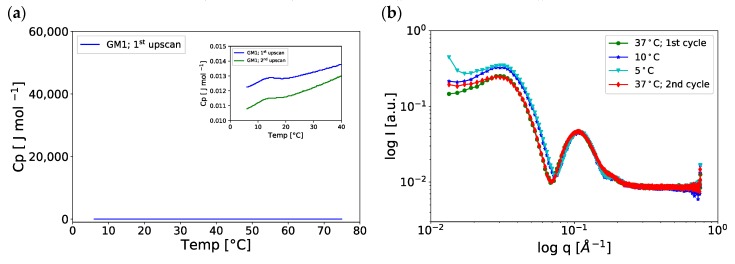
(**a**) DSC thermogram of GM1 in MES buffer at 3 mM concentration, showing no major heat events. Inset: Zoom-in on the small enthalpy effect, approx. 14 °C. The 2nd upscan (green curve) was run after one day of initial 1st upscan (blue curve). (**b**) Temperature studies of GM1. SAXS curves *I(q)* vs. *q* of ganglioside GM1 at 25 mM concentrations, measured at various temperatures. After initial measurements at 37 °C (green curve), the sample was measured at 10 °C (blue) and 5 °C (cyan) before going back to 37 °C (red) again.

From the DSC thermogram in Figure 7a and Figure S6b, we conclude that there are no large enthalpy effects in the temperature range investigated (5‒75 °C). To confirm that the chain melting of GM1 in excess aqueous solution lies below 5 °C, we also performed PTssNMR experiments for GM1 samples at 5 °C (Figure S6a), to be compared with the data obtained for the same sample at 37 °C (Figure 2a). For both temperatures, only an INEPT signal is observed for the whole spectral regime, and there are no visible CP signals for the lipid chain or for the large lipid headgroup. This is consistent with the formation of micelles with a fluid interior and isotropic motion at both temperatures.

We now go back to the DSC data in Figure 7a, where a close inspection of the thermogram shows a low-enthalpy effect at ca. 14 °C (see figure inset). This bump is also seen at the same temperature in the second temperature upscan (Figure 7a—green curve). To further investigate this low-enthalpy temperature-induced change, we performed SAXS at temperatures above and below 14 °C. From the SAXS data in Figure 7b, we conclude that the characteristic minimum in the spectra measured at the lower temperatures (10 °C and 5 °C) is slightly shifted to higher *q* values as compared to spectra measured at 37 °C, indicating a small decrease in the size of the micellar objects when the temperature is reduced. This small-enthalpy change may be associated with conformational changes in the large oligosaccharide ganglioside headgroup moiety, as has previously been proposed by others [46]. To check the reversibility of these temperature-induced changes, the same sample was studied at 37 °C both before and after cooling to 5 °C (Figure 7b). The spectra of these samples were almost identical.

## 3. Discussion

Changes between self-assembled structures can have a huge impact on different cellular processes as changes in structure can influence protein binding, solubilization, and transport processes. It is therefore very important to know if the system contains only one type of structure, or if there are co-existing structures with different properties. The structures that are relevant for the present studies are micelles, vesicles, and lipid discs. The micelles are small assemblies that can dissolve and stabilize insoluble molecules, including proteins, thus offering a transport mechanism in aqueous environments. The micellar assemblies are typically dynamic structures with a relatively fast relaxation time for monomer exchange and a rather short aggregate lifetime [50]. Lipid bilayers, on the other hand, can be infinite in the plane of the bilayers; in biological systems, their major role is to control the transport between different environments inside and outside the cells. In the bilayer, the lateral diffusion in the plane of the membrane is fast, while transmembrane transport processes are generally very slow [50]. The small micellar aggregates have high curvature, while bilayers in vesicles can generally be considered close to planar interfaces at the molecular length scale. For mixed systems of zwitterionic phospholipids and anionic gangliosides, one also needs to consider that the PC-rich vesicles and GM1-rich micelles will have very different surface charge density, which will impact its interactions with proteins and other molecules.

We here show that lipid bilayer membranes co-exist with ganglioside-rich micelles in mixed DOPC-GM1 systems already at relatively low GM1 concentrations. This aspect of the micelle-bilayer co-existence has to be considered in the design and analysis of experiments for mixed-model membrane systems containing gangliosides with large headgroups. Still, this property of the system seems to be neglected in many studies, with some exceptions. Bilayer-micelle co-existence has been shown for model mixtures containing GM1 and ceramides [65]. The co-existence of gel phase bilayers with solid acyl chains and GM1-rich micelles has also been shown by several groups [58,66,67], and in some of these papers the possibility of fluid bilayers-micelle co-existence is also discussed. There are also reports of the co-existence of different types of micelles in mixed ganglioside systems [68]. When incorporated into the bilayer, GM1 molecules may affect both interlamellar interactions as well as the packing and organization of lipids within the bilayer [26,27,28,64,69]. For the DOPC-GM1 model system investigated here, we observed both increased lamellar repeat distance (Figure 5b) and reduced acyl chain mobility (Figure 3c‒g) for the GM1-containing bilayers systems. We note that for the DOPC-GM1 system under the present solution and concentration conditions, the limit where the bilayer membrane will co-exist with other small self-assemblies, like micelles, is lower than 10 mol % GM1. This “saturation” limit will vary with changes in the lipid composition, bilayer phase, and overall lipid concentration [1,31,66].

Although this is not directly investigated here, it is important to point out that the proportions of micelles and vesicles will not only depend on the ratio between the different lipid species, but also on their total concentrations. Consider a sample with a constant DOPC-GM1 ratio. For conditions where the total ganglioside concentration exceeds the CMC, GM1 will be present in DOPC-rich bilayers, in GM1-rich micelles, and as free monomers (maximum concentration given by CMC). If this sample is then diluted, one can reach conditions where micelles are not formed (if the GM1 concentration is below its CMC), and GM1 will then only be found in the bilayer and as monomers in solution. In line with this argument, one can expect that the proportion of micelles to vesicles will decrease with a decreasing overall lipid concentration. The kinetics for ganglioside transfer from micelles to bilayers, starting from nonequilibrium conditions, has been investigated and seems to occur on time scales of minutes to hours [70]. Similar behavior of micelle‒vesicles co-existence is also highly relevant for, e.g., mixtures of bile salts and phospholipids and cholesterol [71,72].

In mixtures that contain several lipid species, it is possible that new types of structures are formed that cannot be obtained from the single components alone. One example of this is the small nonglobular objects observed in samples with a higher GM1 content, which are here interpreted as lipid discs. Similar disc-like structures have previously been shown for other lipid systems containing lipids with large hydrophilic headgroups—for example, lipodiscs formed by PC and PEG-ylated lipids [73,74]. The lipids with large headgroups—gangliosides or PEG-ylated lipids—are then expected to be preferentially located at the curved edges of the discs.

Regarding the biological and clinical relevance of the present results, we have pointed out how changes between different self-assembly structures can impact several fundamental properties of the system. In relation to the example highlighted in the introduction, that ganglioside-containing lipid systems can cause an acceleration in the amyloid aggregation of α-synuclein, we can speculate on some aspects of the system that may indeed influence the interaction between this protein and the lipid assemblies. It has been shown that α-synuclein adsorbs to lipid membranes and micelles with a negative charge [36,75,76,77]. In the adsorbed state, the protein forms an α-helix at the membrane/micelle interface [36,75,78], and membranes covered with proteins are also a trigger for the aggregation process [36,75]. Here, the mode of binding strongly depends on the size and curvature of the lipid assembly: a broken helix is formed for micelles, while for large vesicles the helix is unbroken [79,80]. In a system with co-existing highly charged micelles and planar membranes with low surface charge density, one can foresee an exchange between different protein conformational states, which may also impact the nucleation and aggregation process [36,75,81]. Furthermore, the protein aggregation process strongly depends on the relationship between protein concentration and the total area of charged lipid membranes available [36,75]. Disease-related changes in the content and compositions of gangliosides [9,82,83] may therefore affect protein aggregation processes and the formation of amyloid deposits.

## 4. Materials and Methods

### 4.1. Materials

1,2-dioleoyl-sn-glycero-3-phosphocholine (DOPC) and GM1 ganglioside sodium salt from ovine brains were purchased from Avanti Polar Lipids (Alabaster, AL, USA). 2-(N-morpholino)ethanesulfonic acid, sodium azide and sodium hydroxide, chloroform, and methanol were purchased from Sigma-Aldrich Chemie GmbH (Schnelldorf, Germany). All water was of Millipore quality, produced by MilliQ water filtration system.

### 4.2. Sample Preparation

All the samples investigated were prepared in 10 mM MES buffer, pH 5.5. An appropriate amount of 2-(N-morpholino)ethanesulfonic acid was mixed in water and the pH was adjusted using NaOH solution to achieve a final pH of 5.5. Sodium azide (0.02%) was used in the buffer to avoid fungal contamination.

Samples of ganglioside GM1 and DOPC mixtures at different proportions were prepared as aqueous dispersions with total lipid concentrations of 2‒25 mM, depending on the experimental method (Table 1). For the preparation, we first prepared a molecular mixture of the lipids in a solvent where they were soluble as monomers; here we used a chloroform:methanol ratio of 3:1 (*v*/*v*). The solvent was then evaporated and the sample was placed in a vacuum oven overnight for complete removal of the solvents. The mixed lipid films were then hydrated in 10 mM MES buffer pH 5.5 to get the desired concentration, and dispersed through vortexing. The final solution containing only GM1 was transparent, while all samples containing DOPC appeared milky. This difference indicates aggregates of different sizes in these different samples. These lipid dispersions were then analyzed using different methods.

SAXS, ssNMR, and DLS experiments were performed about 2‒5 h after sample preparation. For SAXS experiments, the samples were contained in screw-tight sandwich cells with mica support and the cells were mounted on a temperature-controlled sample holder in an X-ray device for measurements. For the ssNMR experiments, the samples were loaded in a tight insert (Bruker, Karlsruhe, Germany) and subsequently the insert was placed in an NMR rotor (Bruker, Karlsruhe, Germany) for measurements. For DSC measurements, the sample was prepared one day before the experiment and kept at 4 °C for equilibration prior to DSC measurements. This was done in order to give time for any low-temperature structures to equilibrate. All the experiments except cryo-TEM were performed at 37 °C. For cryo-TEM experiments, the mixtures of GM1 and DOPC at different compositions were further sonicated using a tip sonicator for 20 min, using a pulse sequence of 10 s on/off duty and 75 % amplitude. The lipid dispersions become clear after sonication and the samples were vitrified 2 h after preparation.

### 4.3. Cryogenic Transmission Electron Microscopy (cryo-TEM)

To prepare samples for cryoTEM measurement, 4 μL of each sample were transferred to a glow discharged lacey formvar carbon coated copper grid (PELCO NetMesh, Ted Pella, Redding, California, USA) and vitrified in liquid ethane using an automatic plunge freezer (Leica EM GP, Wetzlar, Germany). The grids were stored in liquid nitrogen until use. A Fischione model 2550 cryo transfer tomography holder was used to transfer the samples into the transmission electron microscope, a JEM-2200FS (JEOL, Peabody, Massachusetts, USA), equipped with an in-column energy filter (Zeiss omega filter, Oberkochen, Germany), which allows for zero-loss imaging. The accelerating voltage was 200 kV and images were recorded with a TVIPS TemCam-F416 digital camera using Serial EM under low-dose conditions. Multiple images were acquired for all cryo-TEM samples at all compositions.

### 4.4. Small-Angle X-Ray Scattering (SAXS)

SAXS studies were performed using an in-house X-ray setup: a SAXSLab Ganesha 300XL instrument (SAXSLAB ApS, Skovlunde, Denmark), equipped with a 2D 300K Pilatus detector (Dectris Ltd., Baden, Switzerland). The scattering intensity (*I*) was recorded as a function of scattering vector *q* in reciprocal Ångström (Å) and is defined as $q=\frac{4\pi sin\theta }{\lambda }$. Here, θ is the scattering angle and λ is the incident X-ray wavelength, which is 1.54 Å in our case. The lamellar *d*-spacing was calculated from the various peak positions of *q* by using the equation $d=\frac{2\pi }{q}$. The sample to detector distance was adjusted based on the *q* range selected. The two-dimensional (2D) scattering pattern recorded by the detector was radially averaged using the software SAXSGui to obtain 1D *I* vs. *q* data. The scattering data were recorded for about 30 to 60 min depending on the sample concentration. All the SAXS experiments were performed in duplicate with different total concentrations and the temperature was controlled using an external circulating water bath to 37 °C. In addition, SAXS experiments were performed for both samples prepared in a buffer (MES pH 5,5) and for samples prepared in pure water.

### 4.5. Differential Scanning Calorimetry (DSC)

The DSC measurements were performed on a VP-DSC calorimeter (Microcal Inc., Northampton, MA, USA), equipped with two cells of 1.2 mL capacity. One cell was used as a reference and the other one for the sample to be studied. Both the reference and the sample were degassed using a Nueva II stirrer before transferring the solution to the cells using a Hamilton syringe. Two upscans were recorded in the temperature range 5‒75 °C with a scanning rate of 90 °C/h. The samples were equilibrated at 5 °C for 30 min before starting the first upscan. The second upscan was recorded after thermostating the sample at 5 °C for 15 h. Data were analyzed using the software Origin from MicroCal, where baseline correction with respect to the reference cell was performed. The DSC experiments were performed in triplicate, with samples prepared both in buffer and in pure water.

### 4.6. Dynamic Light Scattering (DLS)

DLS measurements were performed using a Malvern Zetasizer Nano ZS (Malvern Instruments, Malvern, UK). The samples were diluted using MES buffer to the concentrations provided in Table 1 and poured into a disposable capillary cell for measurements. An initial equilibration time of 120 s was used before each measurement, and the measurements were performed in triplicate. All the DLS experiments were performed in duplicate, with samples prepared both in buffer and in pure water; the measurements were performed at 37 °C.

### 4.7. Polarization Transfer Solid State NMR (PTssNMR)

The PTssNMR method is a set of three different experiments performed on the same sample in a sequential order: DP (direct polarization), CP (cross-polarization), and INEPT (insensitive nuclei enhanced by polarization transfer). PTssNMR experiments were carried out on a Bruker Avance AVII 500 NMR spectrometer. The instrument was equipped with a Bruker E-free 4 mm magic angle spinning (MAS) probe, operated at 5 kHz frequency, and with ^1^H and ^13^C resonance frequencies of 500 and 125 MHz, respectively. The temperature was set to 37 °C and calibrated using methanol. A spectral width of 250 ppm was used and the number of scans per experiment was 2048, with the acquisition time and recycle delay being 0.05 s and 5 s, respectively. This gives a total estimated time of ~9 h for all three experiments. All the PTssNMR experiments were performed in duplicate for samples with different total concentrations. The methylene signal of solid α-glycine at 43.67 ppm was used to calibrate the ^13^C chemical shift scale. Data processing was done using a line broadening of 20 Hz, zero filling from 1597 to 8192 time domain points, Fourier transformation, phase correction, and baseline correction by using an in-house Matlab code partially derived from matNMR.

### 4.8. Diffusion NMR

A pulsed field gradient stimulated echo (PFG-STE) experiment was performed on a Bruker Avance AVII 500 NMR spectrometer using a CP/MAS probe. A ^1^H PFG-STE experiment for ganglioside GM1 lipids was carried out using gradient pulses δ of 2 ms duration and a diffusion delay Δ of 150 ms. The signal was varied with gradient strengths *G* linearly incremented in 32 steps. The decay of ^1^H peak area as a function of the diffusion weighting variable *b* was fitted to a monoexponential decay function:

(1) $$I\left(b\right)=I_{0\;}\exp\left(-\gamma^{2}\;G^{2}\;\delta^{2}\;\left(△-\frac{\delta }{3}\right)D\right)$$

where *D* is the diffusion coefficient and *γ* is the gyromagnetic ratio. The diffusion experiment was performed at 37 °C.

### 4.9. ^31^P NMR

Static ^31^P NMR spectra were acquired using Bruker Avance AVII 500 NMR spectrometer (11.7 T) with a CP/MAS probe. An acquisition time and recycle delay of 0.05 s and 5 s, respectively, were used, together with ^1^H decoupling. A total of 18,000 spectra were acquired using a spectral width of 200 ppm. The ^31^P NMR experiments were performed at 37 °C.

### 4.10. Nnano-ESI-MS

Samples were analyzed on an Orbitrap-Velos Pro mass spectrometer (Thermo Scientific, Waltham, MA, USA) using nano electrospray. Samples (~2 µL) were loaded in disposable emitters and sprayed using negative ionization. Relative quantities were determined by averaging 63 full scan spectra and combining the three first isotopes (if above detection limit) of each lipid species.

## Figures and Tables

**Table 1:** Composition and total lipid concentration of samples used in studies with different complementary techniques.

Sample	Lipid Molar Composition DOPC:GM1	Total Lipid Concentration (mM)				
Name		cryo-TEM	NMR *	SAXS	DLS	DSC
DOPC	100:0	-	25	25	-	-
GM_35_DOPC_65_	65:35	20	25	25	-	-
GM_10_DOPC_90_	90:10	20	25	25	2	-
GM_2.5_DOPC_97.5_	97.5: 2.5	-	25	25	-	-
GM1	0:100	15	15	25 & 5	3	3

* PTssNMR, ^31^P NMR and diffusion NMR.

## Supplementary Materials

Supplementary materials can be found at https://www.mdpi.com/1422-0067/21/1/56/s1.

## Abbreviations

GM	Ganglioside mono-sialylated
DOPC	1,2-dioleoyl-sn-glycero-3-phosphocholine
PTssNMR	Polarization transfer solid state nuclear magnetic resonance
MAS	Magic angle spinning
CP	Cross-polarization
DP	Direct polarization
INEPT	Insensitive nuclei enhanced by polarization transfer
AT	All trans
TG	Trans/gauche
PFG-STE	Pulsed field gradient stimulated echo
TEM	Transmission electron microscopy
SAXS	Small-angle X-ray scattering
ESI MS	Electro spray ionization mass spectrometry
DLS	Dynamic light scattering
DSC	Differential scanning calorimetry
CMC	Critical micellar concentration
