# Supplementary material for: Self-Assembly in Ganglioside‒Phospholipid Systems: The Co-Existence of Vesicles, Micelles, and Discs
Source: Int J Mol Sci. 2019 Dec 20;21(1):56. doi: 10.3390/ijms21010056 (PMC6982371; doi:10.3390/ijms21010056)
Supplement: Supplementary file 1 [file ijms-21-00056-s001.pdf]

Supplementary Materials

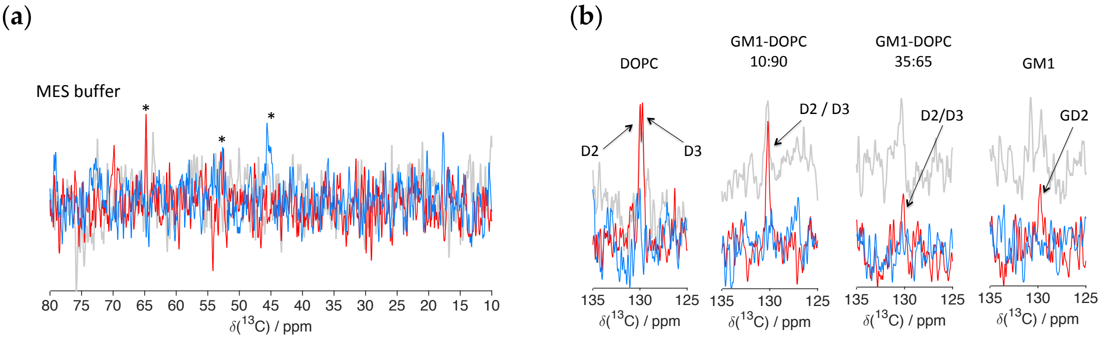

Figure S1. (**a**) The ^{13}C PTssNMR spectra of 10 mM MES buffer pH 5.5. The * indicates peak originate from carbon segments present in the MES. **(b)** PTssNMR ^{13}C spectra of unsaturated double bonds of DOPC and GM1 as labeled in the molecular structure in Figure 1. The individual CP (blue), DP (grey) and INEPT (red) spectra are overlaid and shown for DOPC and GM1 alone and also for the GM1-DOPC mixtures with different molar ratios as indicated in the figure. All experiments were performed at 37 °C.

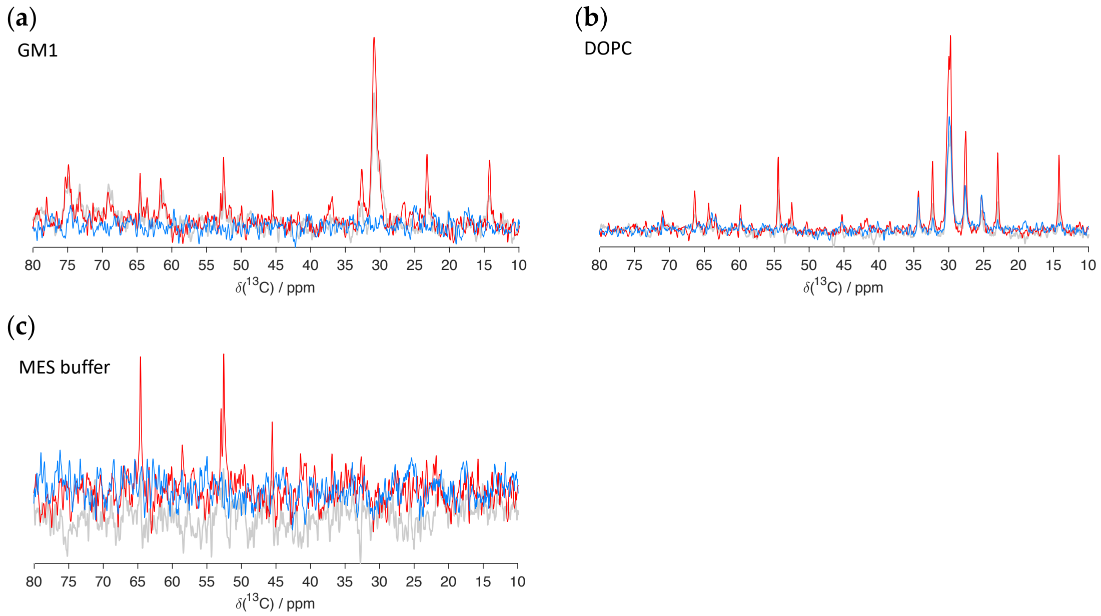

Figure S2. (**a**) PTssNMR ^{13}C spectra of (**a**) GM1 and (**b**) DOPC prepared in MES buffer pH 5.5 and measured at 32 °C. The DP, CP and INEPT set of spectra are overlaid for comparison purpose. In both cases, the spectra show similar trend as was observed when performed those experiments at 37 °C. (**c**) ^{13}C spectra of MES buffer measured at 32 °C.

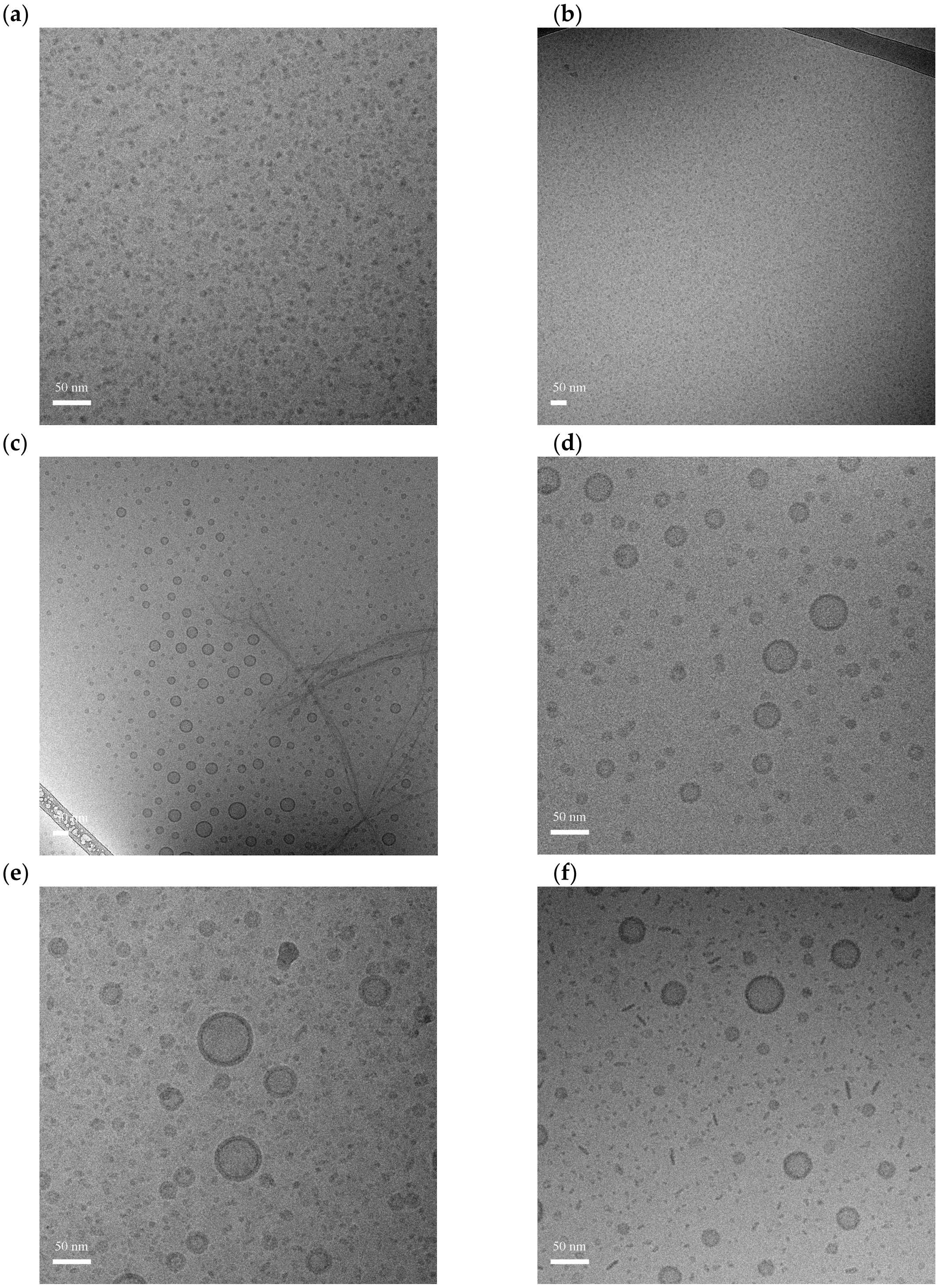

Figure S3. Cryo-TEM images of 15 mM GM1 samples prepared in 10 mM MES buffer pH 5.5 (**a** and **b**). The cryo-TEM images of GM1-DOPC mixtures are presented in **c** and **d** for molar ratios of 10:90 and in **e** and **f** for molar ratios 35:65.

(a) (b)

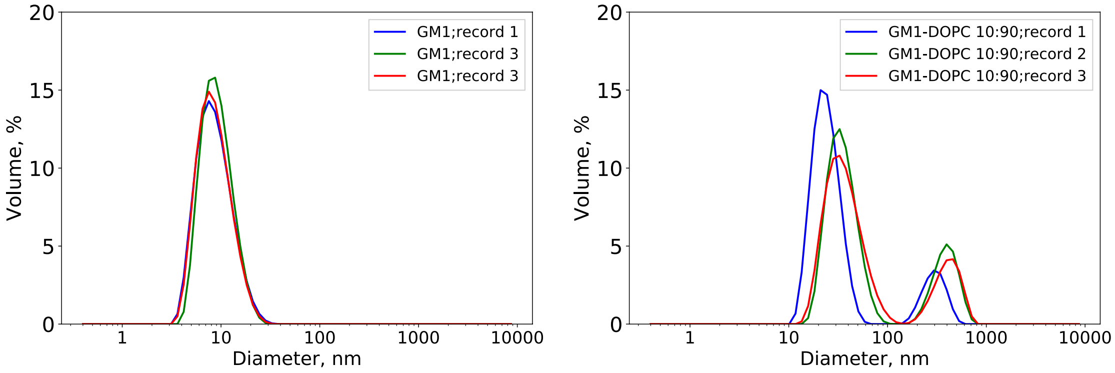

Figure S4. (**a**) DLS measurement of ganglioside GM1 prepared in MES buffer and diluted to 3 mM concentration. The size of the micelles can be approximated from the volume distribution. (**b**) GM1-DOPC 10:90 DLS measurement at dilute concentration of 2 mM in MES buffer showing polydispersity in the system. All the measurements were performed at 37 °C and in triplicate.

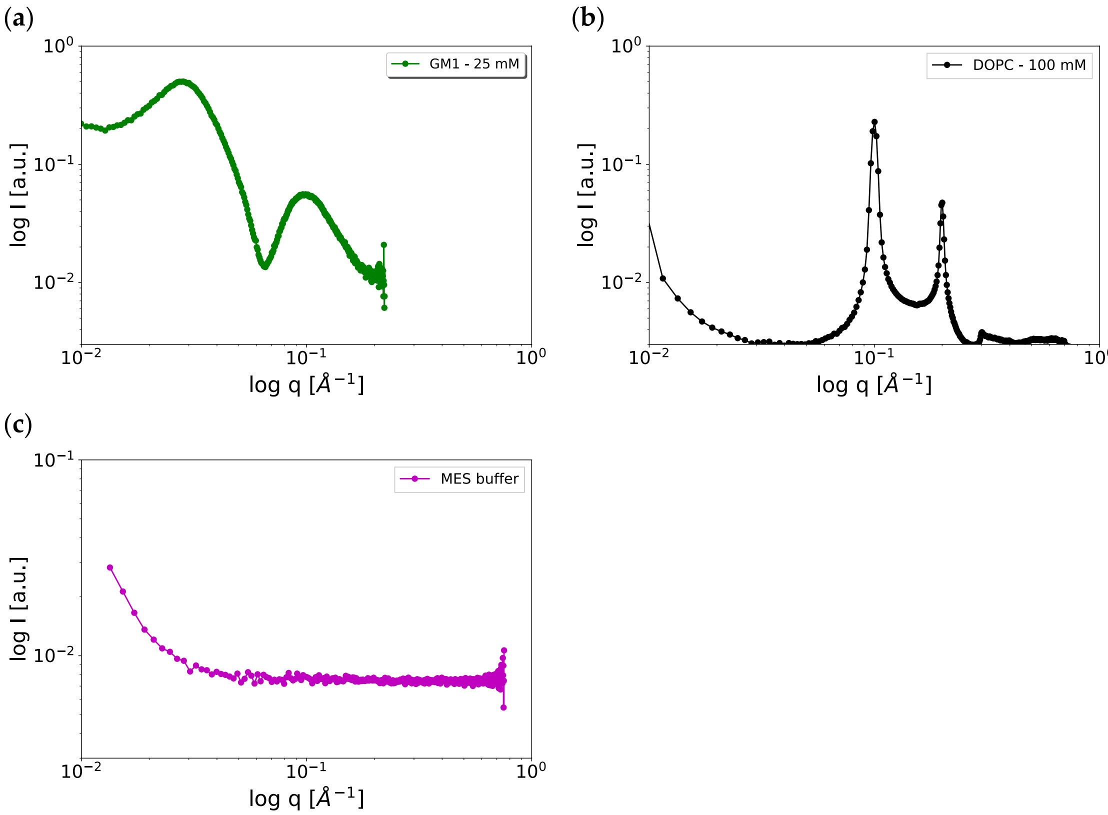

Figure S5. SAXS pattern I vs q of (**a**) ganglioside GM1 at 25 mM and for (**b**) DOPC at 100 mM concentration prepared in pure water (**c**) SAXS profile of MES buffer used to prepare lipid samples. All the measurements were performed at 37 °C using in house SAXSLab Ganesha 300XL instrument.

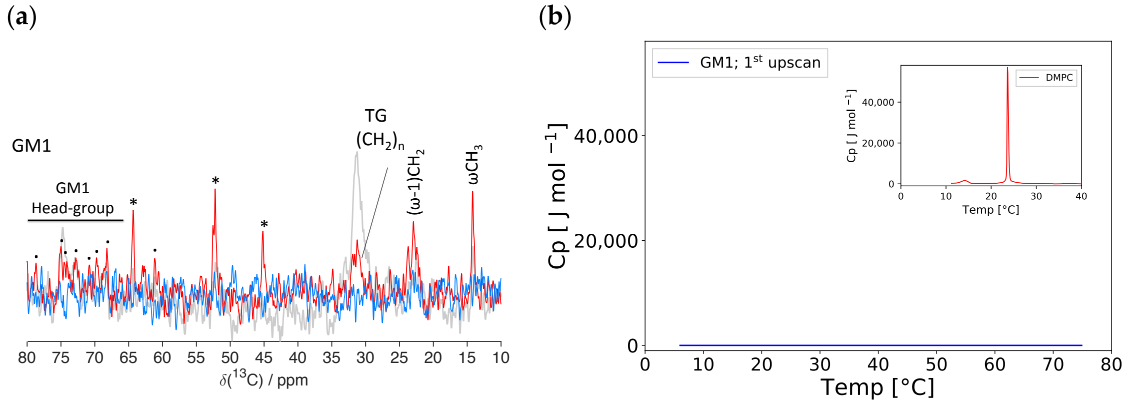

Figure S6. (**a**) PTssNMR ^{13}C spectra of 15 mM GM1 sample prepared in MES buffer and measured at 5 °C. The different molecular segments indicated are according to the label as shown in the Figure 1. The * in the spectra indicates resonance lines originating from the MES buffer used in GM1 sample preparation. The dot (**.**) in the INEPT spectra represent peaks originating from the oligosaccharide head-group of the GM1 molecule. (**b**) DSC thermogram of GM1 and DMPC (presented in the inset for comparison purpose). The pre-transition and main chain transition for the lipid acyl chains are clearly visible with high enthalpy change in case of DMPC. For GM1, presented on the same scale as for DMPC, a flat line is observed, indicating no major enthalpy change occurred during the chosen temperature between 5 to 75 °C.
